# Modular timer networks: abdominal interneurons controlling the chirp and pulse pattern in a cricket calling song

**DOI:** 10.1007/s00359-020-01448-0

**Published:** 2020-10-21

**Authors:** Pedro F. Jacob, Berthold Hedwig

**Affiliations:** 1 Department of Zoology, University of Cambridge, Downing Street, Cambridge, CB2 3EJ UK; 2 Champalimaud Neuroscience Program, Champalimaud Centre for the Unknown, Lisbon, Portugal; 3 Present Address: Centre for Neural Circuits and Behaviour, The University of Oxford, Tinsley Building, Mansfield Road, Oxford, OX1 3SR UK

**Keywords:** Acoustic communication, Central pattern generator, Identified interneurons, Modular network, Timing of rhythms

## Abstract

Chirping male crickets combine a 30 Hz pulse pattern with a 3 Hz chirp pattern to drive the rhythmic opening-closing movements of the front wings for sound production. Lesion experiments suggest two coupled modular timer-networks located along the chain of abdominal ganglia, a network in A3 and A4 generating the pulse pattern, and a network organized along with ganglia A4–A6 controlling the generation of the chirp rhythm. We analyzed neurons of the timer-networks and their synaptic connections by intracellular recordings and staining. We identified neurons spiking in phase with the chirps and pulses, or that are inhibited during the chirps. Neurons share a similar “gestalt”, regarding the position of the cell body, the dendritic arborizations and the contralateral ascending axon. Activating neurons of the pulse-timer network elicits ongoing motor activity driving the generation of pulses; this activity is not structured in the chirp pattern. Activating neurons of the chirp-timer network excites pulse-timer neurons; it drives the generation of chirps and during the chirps the pulse pattern is produced. Our results support the hypothesis that two modular networks along the abdominal ganglion chain control the cricket calling song, a pattern generating network in the mesothoracic ganglion may not be required.

## Introduction

Some of the most complex signal patterns and motor programs in invertebrates are displayed in the context of intraspecific communication. The elaborate acoustic and/or vibratory signals of drosophilids (Mazzoni et al. 2013; Herrnández and Fabre 2016), hemiptera such as cicadas and bugs (Claridge 1985; Fonseca 1991; Virant-Doberlet and Cokl 2004), and orthopteran insects such as acridid grasshoppers (Otte 1970; Bull 1979; Vedenina and von Helversen 2003) and crickets (Alexander 1962; Otte 1992) are crucial for species-specific recognition during mating behaviour. Their evolution has been shaped by sexual selection (Zahavi 1980; Huber and Gerhardt 2002; Ronacher 2019) to produce reliable and species-specific communication signals, which can be stereotypic signal sequences like in a Morse code. The timing of the signalling events is crucial for the receiver’s recognition process (Hennig et al. 2014). Within the nervous system, specialized neuronal networks forming central pattern generators (CPGs) (Delcomyn 1980; Cropper and Weiss 1996; Marder and Calabrese 1996; Selverston 1980, 2010) ensure the precise generation of rhythmic motor activity. The neural basis underlying motor pattern generation for insect communication behaviour with motor activity on different timescales has only occasionally been addressed (Bentley 1969; Simmons 1977; Gramoll 1988; Hennig 1990; Hedwig 1992; Lins and Elsner 1995; Schöneich and Hedwig 2011, 2012; Jacob and Hedwig 2019). Here we take advantage of the singing behaviour in a chirping cricket, which combines rhythmic motor activity on different time scales to produce its typical calling song of chirps and pulses. We characterize the neural mechanism underlying singing activity to understand where and how the two rhythms are generated, and coordinated.

Male crickets attract females using a species-specific calling song. In chirping species such as the bi-spotted field cricket *Gryllus bimaculatus*, sound signals occur over two different time-scales. Chirps are generated with a repetition rate of about 3 Hz and the pulses within the chirps at a rate of about 30 Hz (Alexander 1962) and two different coupled oscillators controlling the rhythms had been proposed (Bentley 1969; Kutsch and Huber 1989). As crickets use their front wings for sound production, the singing network was initially thought to be housed in the mesothoracic ganglion (Huber 1962). Recent lesion experiments in *G. bimaculatus,* however, revealed that the neuronal network underlying the generation of chirps and pulses is organised along with the abdominal nervous system (Pires and Hoy 1992; Hennig and Otto 1996; Schöneich and Hedwig 2011; Jacob and Hedwig 2016). Lesions to the connectives and ganglia of the abdominal nerve cord altered the timing of chirps, pulses, or both in very specific ways and indicated that the posterior ganglia (A4, A5 and A6) control or affect the generation of the chirp pattern while the anterior ganglia (A3 and A4) control the generation of the pulse pattern. This led to the suggestion of modular timer-networks controlling singing motor pattern generation (Jacob and Hedwig 2016). In the third abdominal ganglion (A3) the ascending opener interneuron, which controls the generation of sound pulses has been identified. The neuron however does not control the chirp rhythm, this rather must be imposed by some higher interneurons which generate the chirp pattern (Schöneich and Hedwig 2012). To analyse the cellular and network properties of the chirp-timer and pulse-timer networks in more detail, we explored the abdominal ganglia with intracellular recordings and staining during fictive singing of male *G. bimaculatus*. We aimed to reveal how neurons and neural networks are organised to generate and coordinate the two motor rhythms underlying cricket singing behaviour.

## Materials and methods

### Animals

Experiments were performed on a total of 54 adult male crickets (*Gryllus bimaculatus* DeGeer). Males were kept individually in plastic containers at 26–28 ºC with a 12 h light:dark cycle and were used from 7 to 21 days post-ecdysis. A mixture of protein-rich food and water was provided ad-libitum. Experiments were carried out at 23–24 ºC and complied with the principles of Laboratory Animal Care (ASAB Ethics Committee 1997).

### Dissection and pharmacological brain stimulation

Prior to experiments crickets were cooled down, and were placed dorsal side up on a Plasticine™ block by restraining all legs with metal clamps. The head was waxed to a metal holder and opened to expose the brain. We accessed the central nervous system by a dorsal midline incision along the abdomen and thorax, peripheral nerves to the thoracic and abdominal ganglia were cut, except for the mesothoracic wing nerve 3A (meso-Nv3A), and the cercal nerves; see Jacob and Hedwig (2015) for details.

Exposed nervous tissue was rinsed with saline, in mM: NaCl 140; KCl 10; CaCl_2_ 7; NaHCO_3_ 8; MgCl_2_ 1; TES 5; D-trehalose dehydrate 4, adjusted to pH 7.4. To elicit fictive singing, glass capillaries filled with eserine salicylate (10^− 2^ mol l^− 1^; Sigma-Aldrich, St Louis, MO, USA) in saline were inserted into the ventral protocerebrum and the solution was pressure injected (Pneumatic PicoPump PV820, WPI, Sarasota, FL, USA). See Wenzel and Hedwig (1999) and Schöneich and Hedwig (2012) for details.

The singing motor pattern was recorded from the meso-Nv3A, (Jacob and Hedwig 2015), which contains the axons of the front-wing opener and closer motoneurons (MN) (Kutsch and Huber 1989), further referred to as opener and closer MN. We used a double-hook electrode made from 100 µm platinum wire and amplified the signal with a differential AC amplifier (Model 1700; A-M Systems, Sequim, WA, USA). During singing the nerve recording reliably showed the rhythmically alternating spike activity of the MN. We identified the opener MNs as the ones that were always activated first and followed by the activity of the closer MNs. Compared to normal singing, the opener-closer interval corresponds to the silent opening movement and the closer-opener interval to the sonorous closing movement of the wings.

### Intracellular recordings of singing-CPG neurons in the abdominal ganglia

The relevant abdominal ganglia were stabilized between a dorsal stainless-steel platform and a ventral tungsten ring. To record neurites located in the ventral neuropil, the ganglion was flipped with the ventral side up. Microcapillaries were pulled (DMZ-Universal Puller, Zeitz-Instruments, Martinsried, Germany) from thick-walled borosilicate glass tubes (ID 0.58 mm, OD 1.0 mm; Hilgenberg GmbH, Malsfeld, Germany). Intracellular recordings were made in bridge mode (SEC10-05LX amplifier; NPI, Tamm, Germany) and sampled at 40 kHz per channel (Micro1401 mk II, CED, Cambridge, UK).

The neurons identified in this work were considered to belong to the singing-CPG according to criteria established by Marder and Calabrese (1996). (1) The neurons are active in time with the singing motor pattern. (2) The neurons initiate, terminate, or change the singing motor activity. (3) The neurons have direct or oligosynaptic connections with the relevant motoneurons.

### Neuron morphology

Fluorescent dyes were iontophoretically injected into recorded neurons by constant hyperpolarizing current injection with 0.5–5 nA, up to 20 min for 0.5% Alexa 568 hydrazide sodium salt (Molecular Probes Inc., Eugene, OR, USA) and 1% Lucifer Yellow (Sigma-Aldrich, St Louis, MO, USA) or by depolarizing pulses with 2–3 nA, 200 ms duration, 3 Hz, up to 60 min for 2–4% neurobiotin (Vector Laboratories, Burlingame, CA, USA). The electrode shaft was backfilled with 2 M potassium acetate for Alexa 568 and neurobiotin, and 1 M lithium chloride for Lucifer Yellow. Microelectrodes had final resistances 80–140 MΩ. Histological processing for fluorescent dyes and neurobiotin staining followed conventional protocols (Schöneich et al. 2011). The whole-mount preparations were scanned with a confocal laser-scanning microscope (Leica SP5, Wetzlar, Germany), and the morphology of neurons reconstructed from the image stacks using the Simple Neurite Tracer plugin in ImageJ (National Institutes of Health, Bethesda, MD, USA). All neurons presented were stained with neurobiotin, unless otherwise stated. Since some neurons were dye-coupled, only a short 10 min protocol of neurobiotin injection was performed. This was sufficient to stain the neuron up to the metathoracic ganglion (T3) but did not stain the dye-coupled neuron. Morphological descriptions are based on the characteristic features for each type of identified interneurons, as details can vary from animal to animal. “Ipsilateral” and “contralateral” are used in relation to a neuron’s cell body.

### Data analysis

Neurophysiological recordings were analyzed with CED Spike2 software (CED, Cambridge, UK) and with NEUROLAB (Knepper and Hedwig 1997).

The membrane potential, the spike rate of the interneurons and the meso-Nv3A activity were averaged over the period of the chirp-cycle and the period of the opener MN cycle by phase-dependent processing in NEUROLAB; i.e. the mean cycle duration was calculated and all responses were normalized to the mean. The wing-opener cycle was defined by the start of two consecutive opener bursts within a chirp. The chirp cycle was defined by the start of the first opener burst in subsequent chirps; only chirps with the same number of pulses were analysed.

Normally distributed data are given as $\bar{x}$± SD; when normality tests failed the median and interquartile range (IQR: 25th percentile/75th percentile) is presented. In pooled data sets, each contributing animal is equally represented (*N*: number of crickets; *n*: number of chirps and pulses; AP: number of spikes). For statistical analysis, we used GraphPad Prism 6 (GraphPad Software, Inc., La Jolla, CA, USA).

### Characterisation of interneurons

Interneurons of the singing-CPG are characterized based on the latency between their spiking activity and the opener burst (Schöneich and Hedwig 2011, 2012). The organisation of the singing network along with the abdominal ganglia, however, comes with a considerable increase in latency between the interneuron and the motoneuron activity. Therefore additional features were used for the functional characterisation of interneurons originating in the posterior ganglia. Interneurons generating a phase-coupled depolarisation before the opener burst and an inhibition before the closer burst were classified as opener-interneurons; these interneurons ended a chirp with an inhibition. Interneurons classified as closer-interneurons were depolarised in a phase-coupled manner before the wing-closer burst and were hyperpolarised or did not fire before the wing-opener activity; they were depolarised at the end of a chirp.

Furthermore, an interneuron was assigned to the pulse-timer network if the time course of its rhythmic membrane depolarisations and spiking activity closely matched either the opener burst or the closer burst; and/or if transient perturbations of its activity by current injection generated additional singing motor activity. According to these criteria, two previously identified interneurons in the abdominal ganglion A3, the ascending opener-interneuron (A3–AO) and a non-identified closer-interneuron (Schöneich and Hedwig 2012) can be classified as pulse-timer interneurons. An interneuron was assigned to the chirp-timer network if its activity was coupled to the chirp pattern and if current injection elicited or modulated the chirp pattern. Corresponding to previous conventions, we label the unfused abdominal ganglia A3–A6.

## Results

Interneurons coupled to the generation of the pulse pattern and chirp pattern were recorded in the chain of abdominal ganglia and will be referred to as “singing-interneurons”. We label the neurons based on the ganglion which houses the cell body, and on their spike activity in relation to the song pattern.

## Structure of singing-interneurons in the unfused abdominal ganglia

**Fig. 1:**
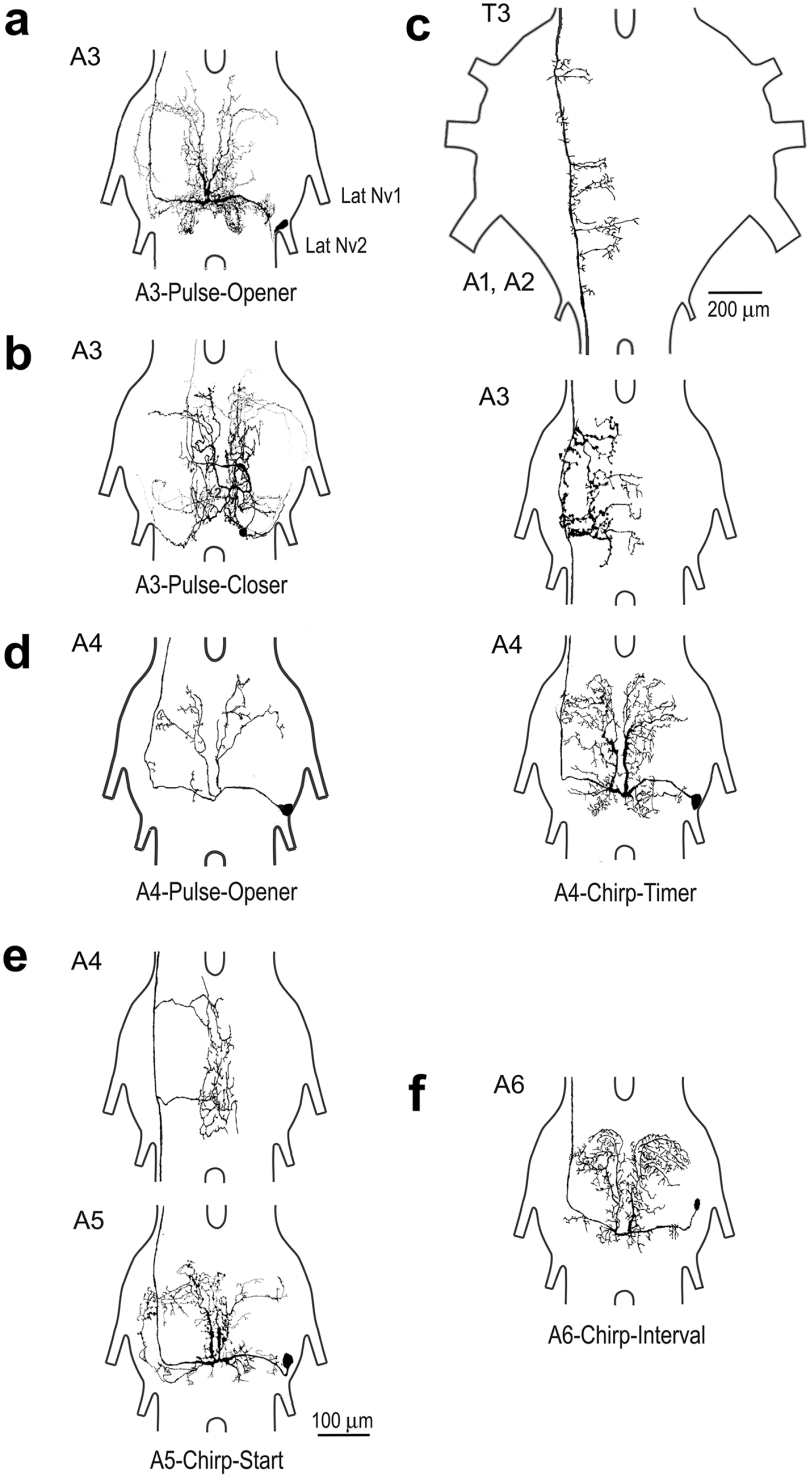
Structure of singing-interneurons with cell bodies located in the abdominal ganglia. All neurons show a lateral cell body, extensive symmetrical dendrites in the dorsal neuropil and a contralateral axon projecting anteriorly, with medial collaterals, if labelled. **a** *A3-Pulse-Opener* interneuron, with cell body and dendrites located in A3 and axon projecting towards the T3 ganglion, dye coupling between bilateral neurons. **b** The *A3-Pulse-Closer* neuron, with complex dendrites in A3 and an ascending axon. **c** The *A4-Chirp-Timer* neuron in ganglion A4 with an axon projecting through A3 and T3, dye-coupling occurred between bilateral neurons. **d** *A4-Pulse-Opener* interneuron, with typically sparse dendrites. **e** *A5-Chirp-Start* interneuron in A5, the axon projects at least through A4. **f** *A6-Chirp-Interval* neuron with cell body and dendrites stained

We identified 6 types of singing-interneurons in the unfused abdominal ganglia, their structure revealed a similar “gestalt” with common features and characteristics (Fig. 1).

In most singing-interneurons the cell body was located in a ventral, posterior lateral position between the roots of lateral Nv1 and Nv2 (Fig. 1a,c–e). The cell body of the *A3-Pulse-Closer* neuron was more median (Fig. 1b), and the cell body of the *A6-Chirp-Interval* neuron was located anterior to the root of lateral Nv1 (Fig. 1f). A primary neurite projected medially and dorsal towards the midline of the corresponding ganglion. Here each neuron symmetrically gave off extensive elaborate dendrites to the anterior dorsal neuropil, while smaller dendrites extended posteriorly from the main neurite. An axon branched off from the main neurite at the midline and always projected towards the more anterior ganglia in the contralateral connective, i.e. relative to the cell body. The axon generally took a very lateral position, however in case of the *A3-Pulse-Closer* it entered the connective in a very medial position (Fig. 1b). Axons gave off elaborate collaterals projecting towards and across the midline of the abdominal or thoracic ganglia (Fig. 1c,e). We did not encounter singing-interneurons with axons projecting posteriorly.

## Interneurons with activity coupled to the pulse pattern

In ganglion A3 and A4 we identified singing-interneurons with activity patterns closely coupled to the pulses.

### The *A3-Pulse-Opener* interneuron

The *A3-Pulse-Opener* interneuron was recorded in 6 males, its structure and functional properties have been described by Schöneich and Hedwig (2011, 2012) and Jacob and Hedwig (2019). We refer to this neuron as the *A3-Pulse-Opener* and give a brief account as it is most relevant for this study. The dendrites of the *A3-Pulse-Opener* are located in A3 (Fig. 1a), its axon projects anterior with medial collaterals in all thoracic ganglia. Dye coupling between bilateral sibling neurons occurred for labelling with neurobiotin (Schöneich and Hedwig 2011, 2012).

**Fig. 2:**
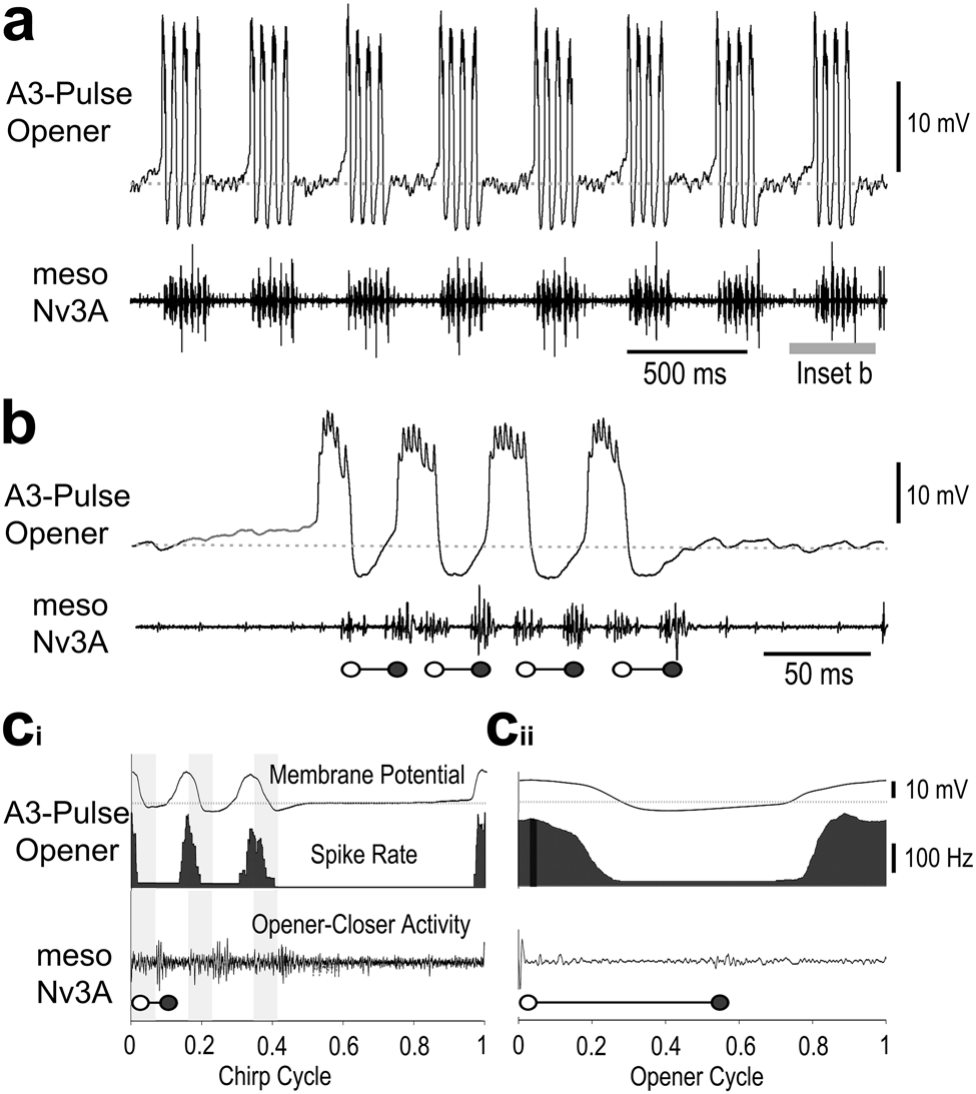
Activity of the *A3-Pulse-Opener* neuron. **a, b** Rhythmic membrane potential depolarisations occur coupled to singing activity. High frequency bursts of spikes precede the opener MN activity (open circles), and hyperpolarisation precedes closer activity (closed circles) recorded from meso-Nv3A. Right after a chirp, activity terminates with a hyperpolarisation, followed by a ramp depolarisation but no spiking activity in the interchirp intervals. **c**_**i**_, **c**_**ii**_ Phase diagrams with the average membrane potential (top), the average spike rate (middle) and the average meso-Nv3A activity (bottom) for the chirp cycle (**c**_**i**_) and opener cycle (**c**_**ii**_). Grey shaded bars indicate opener MN activity, grey dashed line represents the resting membrane potential. *N* = 6 crickets, *n* = 60 chirps/180 pulses, AP = 595

The rhythmic activity of the *A3-Pulse-Opener* neuron was tightly coupled to the pulse pattern, a pronounced depolarisation of 17.5 mV with a high frequency burst of 4–6 spikes preceded each opener activity by 10.4 ± 1.2 ms, followed by a pronounced inhibition of 5–7 mV preceding the closer activity (Fig. 2a,b). No spike activity occurred in the interchirp intervals, about 42 ± 6.4 ms before the opener activity at the beginning of a chirp a gradual ramp-like depolarisation developed, reaching an amplitude of 6 ± 0.5 mV and leading to a rapid pronounced membrane depolarisation of 10–13 mV before of the first MN opener burst (Fig. 2b, c). After 14–19 ms, the membrane potential rapidly dropped, the neuron became hyperpolarised by 5–8 mV, and then depolarised again and generated the next opener burst. Depolarising current injection into the *A3-Pulse-Opener* neuron reliably elicited rhythmic membrane potential oscillations corresponding the pulse pattern, and reset the chirp pattern. Therefore, this interneuron is regarded as an element of the singing CPG (Schöneich and Hedwig 2011, 2012; Jacob and Hedwig 2019). The phase diagram over the chirp cycle shows the tight coupling of the membrane potential oscillations and the spike activity to the pulse pattern (Fig. 2c_i_). In the phase diagram of the opener cycle, the mean spike activity is about 250 AP/s occurring at phase 0.8, i.e. before the opener MN activity (Fig. 2c_ii_) and terminating during the opener activity at phase 0.15.

### The *A3-Pulse-Closer* interneuron

The *A3-Pulse-Closer* was recorded in its main neurite and labelled with Alexa 568 in 5 males, in each staining only one cell body was observed (Fig. 1b). The neuron had a ventral located cell body posterior to the position of lateral Nv2. The main neurite ran ventrally and anteriorly, it give off an axon and a large neurite, which projected dorsally. The neurite subdivided into complex symmetrical dendrites, which on both sides of the ganglion extended towards anterior; dendrites were more profuse at the side of the cell body. The axon had a median position in the contralateral connective, its further projections were not revealed.

**Fig. 3:**
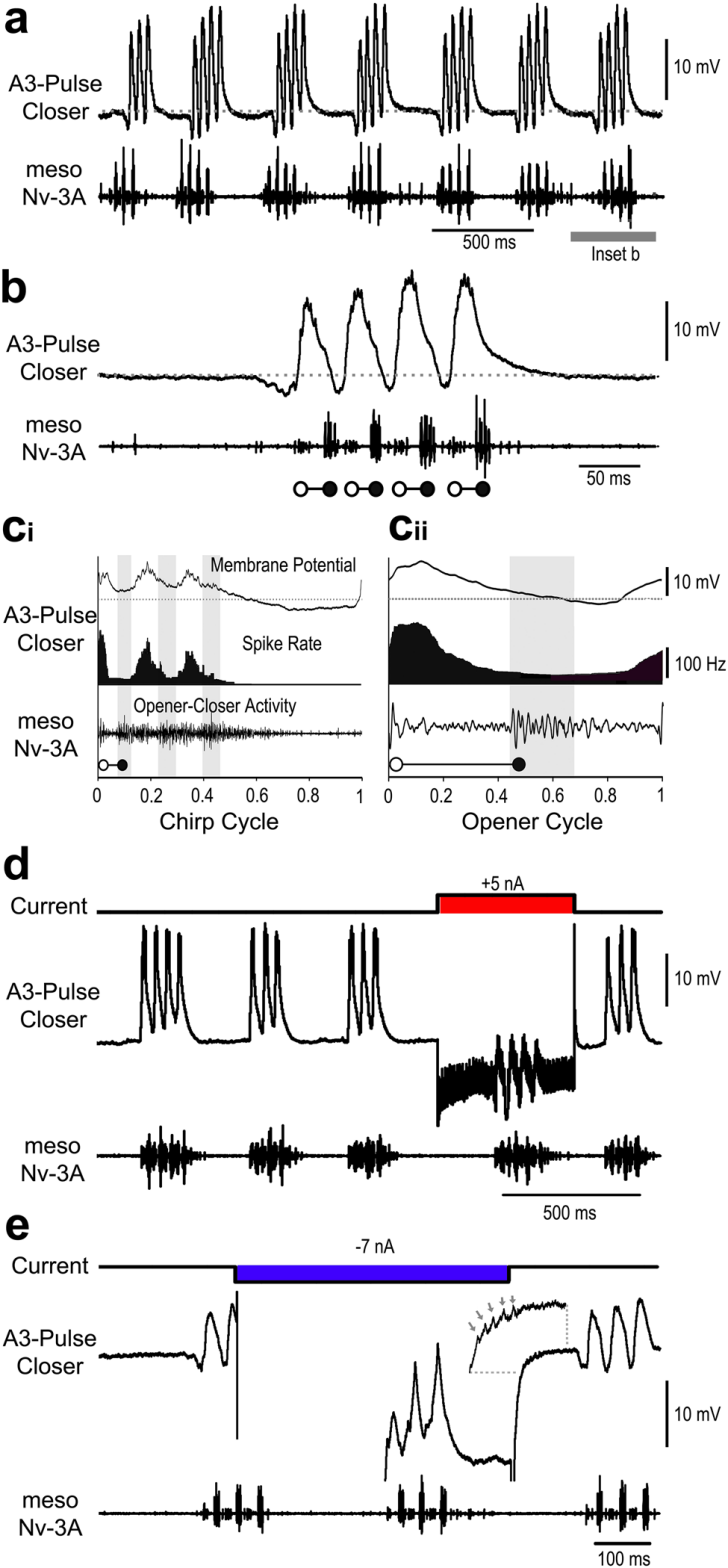
Activity the *A3-Pulse-Closer* neuron. **a, b** Rhythmic membrane potential depolarisations are coupled to singing activity. Hyperpolarisation precedes the opener MN activity (open circles) and high frequency bursts of spikes precede the closer MN activity (closed circles). At the end of a chirp activity terminates with a gradual decline to the resting membrane potential. No spiking activity occurs in the interchirp intervals. **c**_**i**_, **c**_**ii**_ Phase diagrams with the average membrane potential (top), the average spike rate (middle) and the average meso-Nv3A activity (bottom) for the chirp cycle (**c**_**i**_) and opener cycle (**c**_**ii**_). Grey shaded bars indicate closer MN activity, grey dashed line represents the resting membrane potential. *N* = 5 crickets, *n* = 50 chirps/200 pulses, AP = 800. **d** Current injection into the *A3-Pulse-Closer* neuron with 5nA for 500 ms extends the ongoing chirp period. **e** Upon release from inhibition, a postinhibitory rebound generates spike activity. Inset shows spikes (arrows) in the rising phase of the rebound

During fictive singing the *A3-Pulse-Closer* showed rhythmic membrane potential oscillations coupled to the pulse pattern (Fig. 3a, b). At the very start of a chirp the neuron was hyperpolarised by 2.6 ± 1 mV preceding the first opener activity in meso-Nv3A; this was followed by a pronounced depolarisation of 11 ± 1.1 mV with 4–6 spikes, preceding the closer activity by 14.6 ± 1.2 ms (*N* = 5; *n* = 320) (Fig. 3b, c). The peak amplitude of the depolarisations increased over a chirp, while the inhibition preceding the opener activity gradually decreased to 1.5 ± 1 mV for the last pulse. After a chirp the membrane potential gradually declined to the resting level, no spikes occurred in the interchirp interval. The coupling of the spike activity to the singing motor activity is revealed in phase histograms (Fig. 3c_i_,_ii_), which were calculated for chirps with 3 pulses. Over a chirp spikes occurred in phase with the opener and preceding the closer activity. The peak activity gradually decreased from 162 Hz in phase with the first pulse to a broad peak of 97 Hz in phase with the third pulse. With the end of the motoneuron activity at phase 0.5 the spike activity reached its minimum while the membrane potential reached its minimum at 0.8 just before the generation of the next burst. Spike activity over the opener cycle confirmed the timing of the depolarisation and spike activity coupled to the closer activity with a discharge rate reaching 212 Hz.

Depolarising current injection (5 nA, 500 ms) transiently increased the chirp period, from 394 ms to 495 ms when the current pulse was applied (Fig. 3d, *n* = 3). Hyperpolarising current (− 7 nA, 500 ms) had no impact, it, however, elicited a subsequent rebound depolarisation combined with spike activity (Fig. 3e). We also recorded two local closer neurons in A3 with highly similar activity patterns to the *A3-Pulse-Closer*, we do not report details as we only obtained weak morphological data.

### The *A4-Pulse-Opener* interneuron

The *A4-Pulse-Opener* neuron was recorded 14 times and stained in 8 animals with either Alexa 568 or LY. From the ventral lateral cell body the primary neurite ran dorsally toward the midline and gave off two anteriorly projecting main dendrites with a consistently sparse branching pattern (Fig. 1d). The contralateral axon ascended laterally in the connective, its projections could not be revealed. All staining’s labelled only one cell body, dye coupling was not observed.

**Fig. 4:**
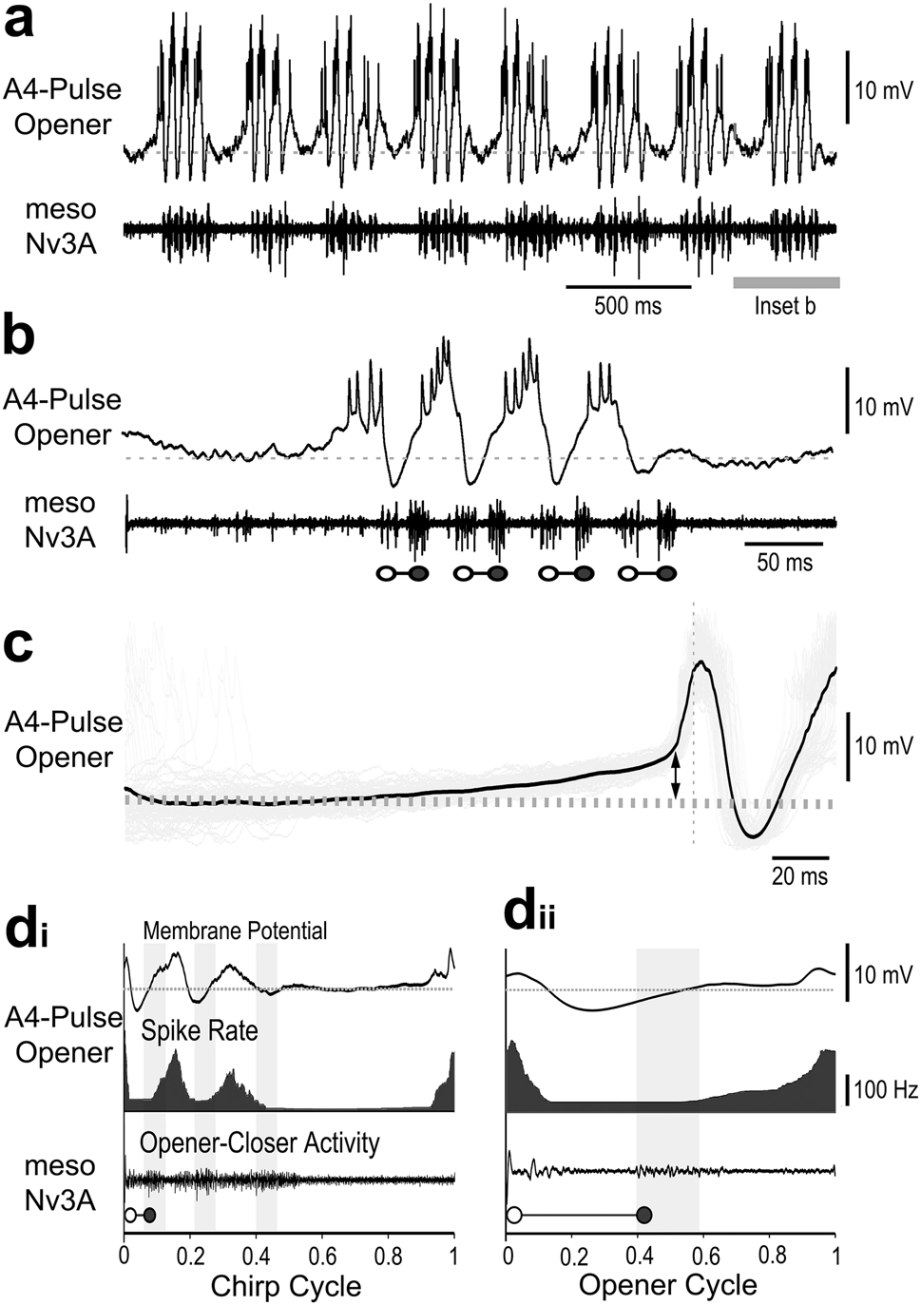
Activity of the *A4-Pulse-Opener*. **a** During fictive singing the *A4-Pulse-Opener* is rhythmically activated during the chirps. **b** Recording shows a gradual ramp-depolarisation at the start of a chirp, bursts of spikes precede and the opener MN activity (open circles) and a pronounced hyperpolarisation precedes the closer MN activity (close circles). Grey dashed line indicates the resting membrane potential. **c** Ramp depolarisation at the start of a chirp leading to the first *A4-Pulse-Opener* spike, indicated by vertical dashed line. Double arrow indicates the peak amplitude of the ramp depolarisation. The black line represents the average and the grey shades the raw signals, respectively. *N* = 14 crickets, *n* = 140 chirps/105 pulses. **d**_**i**_, **d**_**ii**_ Phase diagrams with the average membrane potential of the *A4-Pulse-Opener* (top), its average spike rate (middle) and the averaged meso-Nv3A activity (bottom) for the chirp cycle (**d**_**i**_) and opener cycle (**d**_**ii**_). Vertical grey bars indicate timing of closer MN activity, and grey dashed line the resting membrane potential. *N* = 14 crickets, *n* = 140 chirps/420 pulses, AP = 1540

Similar to the *A3-Pulse-Opener* the rhythmic membrane potential oscillations and spike activity of the *A4-Pulse-Opener* were tightly coupled to the fictive pulse pattern (Fig. 4a). In the chirp interval, a gradual ramp depolarisation started 81 ± 33 ms before each chirp and reached an amplitude of 4.3 ± 1.9 mV (Fig. 4c), the interneuron then depolarised by 14–18 mV and generated a burst of 3–6 spikes preceding the opener MN activity by 17.9 ± 1.9 ms (*N *= 14, *n * = 455) (Fig. 4b,c). Subsequently, the membrane potential dropped to 2–5 mV below the resting potential before the following closer MN activity; thereafter the next depolarisation and burst potential before the following closer MN activity; thereafter the next depolarisation and burst of spikes occurred. At the end of a chirp the final inhibition was followed by a graded subthreshold depolarisation, not coupled to any motor activity (Fig. 4b). In comparison to the *A3-Pulse-Opener*, the depolarisation and repolarisation phases of the *A4-Pulse-Opener* were more gradual.

The phase diagrams of *A4-Pulse-Opener* activity over the chirp cycle (Fig. 4d_i_), show the gradual depolarisation and increase in spike activity towards the end of the chirp interval. The peak of the depolarisation and spike activity occurred before the onset of the opener activity at phase 0.9 with about 260 Hz, respectively. The membrane potential oscillations and spiking activity were coupled to the rhythmic opener activity in meso-Nv3A. The phase diagram over the opener cycle (Fig. 4d_ii_) revealed that the *A4-Pulse-Opener* spike activity started before the opener burst at phase 0.6, followed by an increase of the spike rate to about 210–230 Hz at phase 0.9 just before the onset of the opener activity.

Due to the long latencies of about 17 ms a considerable time shift occurred to the generation of the subsequent opener burst. The phase values obtained, therefore, need careful interpretation, and do not indicate a coupling to the motor activity in phase with the neuronal activity. Interneuron activity in phase with the closer activity, therefore, actually preceded the opener activity.

**Fig. 5:**
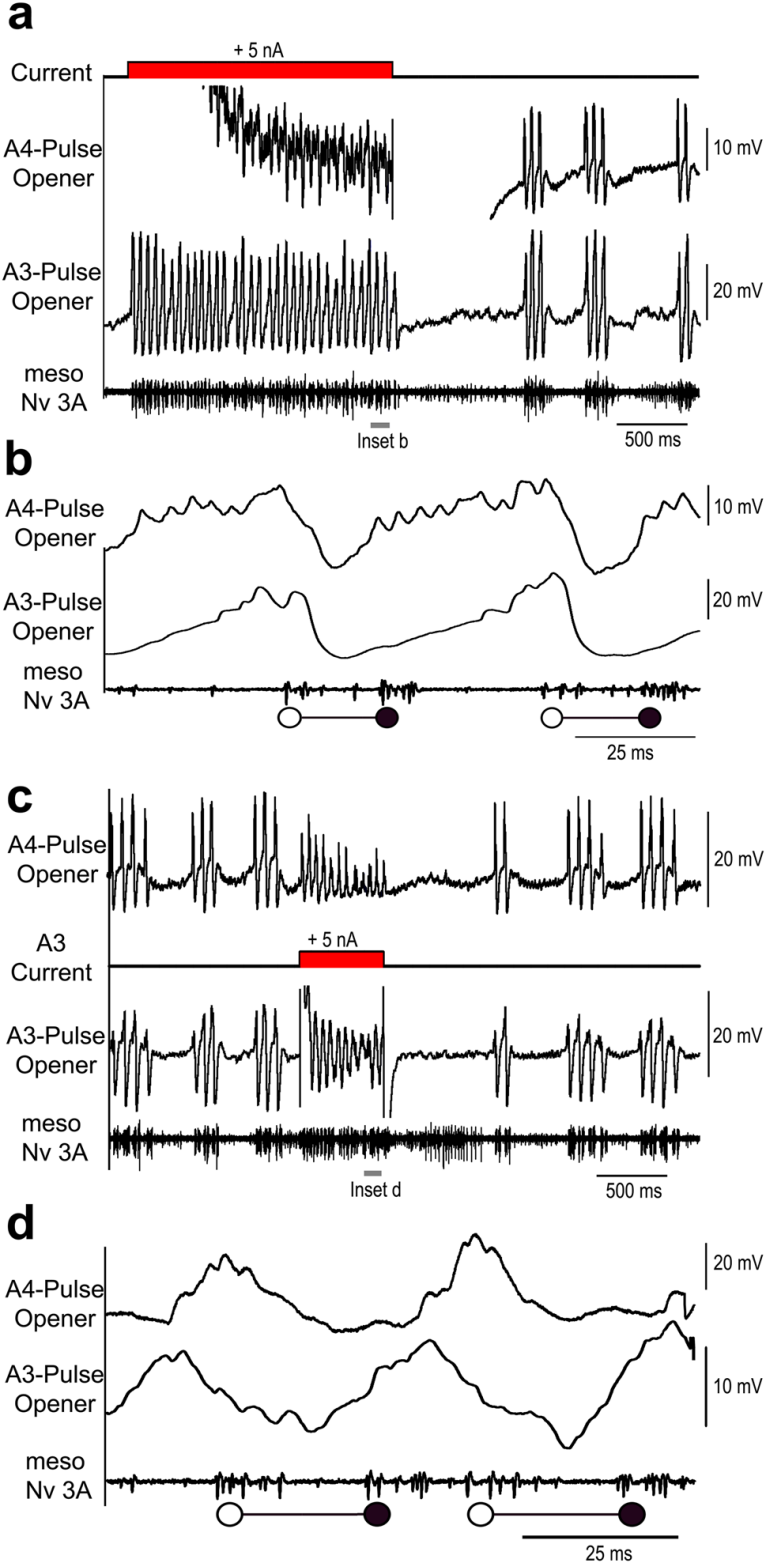
Paired intracellular recordings of the *A4-Pulse-Opener* and the *A3-Pulse-Opener.* **a** Depolarising current injection with 5 nA for 1.8 s in the *A4-Pulse-Opener* drives rhythmic oscillations in in the *A3-Pulse-Opener* and the opener and closer MNs for the duration of the pulse. The chirp pattern resumes after current injection. **b** The recording at high resolution does not reveal synaptic coupling between the *A3-Pulse-Opener* and the *A4-Pulse-Opener*. **c** Depolarising current injection with 5 nA for 600 ms in the *A3-Pulse-Opener* drives rhythmic oscillations in the *A4-Pulse-Opener* and the opener-closer cycles for the duration of the pulse. The chirp pattern resumes after current injection. **d** The recording at high resolution does not reveal synaptic coupling between the interneurons. It reveals a shift in the timing of the depolarisations of both interneurons

We analysed the functional properties of the neuron by paired recordings (*n* = 4) of the *A4-Pulse-Opener* and the *A3-Pulse-Opener* (Fig. 5). Depolarising current injection (5 nA, 1.8 s) into the main neurite of the *A4-Pulse-Opener* altered the ongoing chirp pattern and elicited continuous rhythmic membrane potential oscillations and cycles of opener-closer motor activity (Fig. 5a). The current pulse also elicited rhythmic activity in the *A3-Pulse-Opener* with the membrane depolarisations of both neurons occurring almost in synchrony and preceding the start of the opener MN burst (Fig. 5b). Checking the recordings with high temporal resolution and spike triggered averaging of the *A3-Pulse-Opener* activity did not reveal any EPSPs elicited by the *A4-Pulse-Opener* spikes. The similar membrane potential changes rather indicate a common driving input. The effect of depolarising the *A4-Pulse-Opener* indicates that this interneuron is part of the singing-CPG network that controls the generation of the pulse pattern.

Also current injection (5 nA, 600 ms) in the *A3-Pulse-Opener* altered the motor pattern. It elicited rhythmic activity in the pulse pattern in the *A3-Pulse-Opener*, and also subthreshold rhythmic depolarisations in the *A4-Pulse-Opener* (Fig. 5c,d), which however occurred with a latency of 9.4 ± 2.3 ms (*N *  = 3; *n * = 90) after the peak of the *A3-Pulse-Opener* activity. As a consequence, only the activity of the *A3-Pulse-Opener* preceded the opener MN activity, while the activity of the *A4-Pulse-Opener* was shifted, and occurred in synchrony with the opener activity.

The paired recordings of the *A3-Pulse-Opener* and *A4-Pulse-Opener* allowed to compare the duration of the fictive singing pulse period, measured by the closer MN period, and the period of the depolarisation of the interneurons (*N* = 4). This analysis was performed to check if the rhythmic depolarisations generated by the two interneurons matched the final motor pattern. The average period of the closer MN activity was 49.9 ± 3.4 ms, for the rhythmic depolarisations of the *A3-Pulse-Opener* it was 49.9 ± 3.3 ms and for the *A4-Pulse-Opener* it was 48 ± 2.9 ms. In all four animals analysed, the average period of the MN closer burst was more similar to the period of the *A3-Pulse-Opener* than to the *A4-Pulse-Opener* depolarisations.

## Interneurons with activity coupled to the chirp pattern

### The A4-Chirp-Timer and A5-Chirp-Timer interneurons

In the A4 and A5 ganglion we encountered two interneurons, with highly similar activity patterns coupled to the chirp rhythms, with characteristics of chirp-timer neurons. The *A4-Chirp-Timer* was recorded 6 times, stained 5 times with neurobiotin and once with Alexa 568. It showed extensive symmetrical dendritic ramifications in the A4 ganglion (Fig. 1c), highly similar to the branching pattern of the *A3-Pulse-Opener* and the *A4-Pulse-Opener*. Its axon projected anteriorly in a very lateral position in the connective sending multiple collaterals towards and across the midline of the A3 ganglion. In the metathoracic ganglion (T3) the ramifications occurred mainly in the posterior region and the A1 neuromere and were less elaborate (Fig. 1c). The axon was stained up to T2, but further details could not be revealed. Two staining with neurobiotin demonstrated dye-coupling with a mirror-image sibling neuron on the contralateral side.

**Fig. 6:**
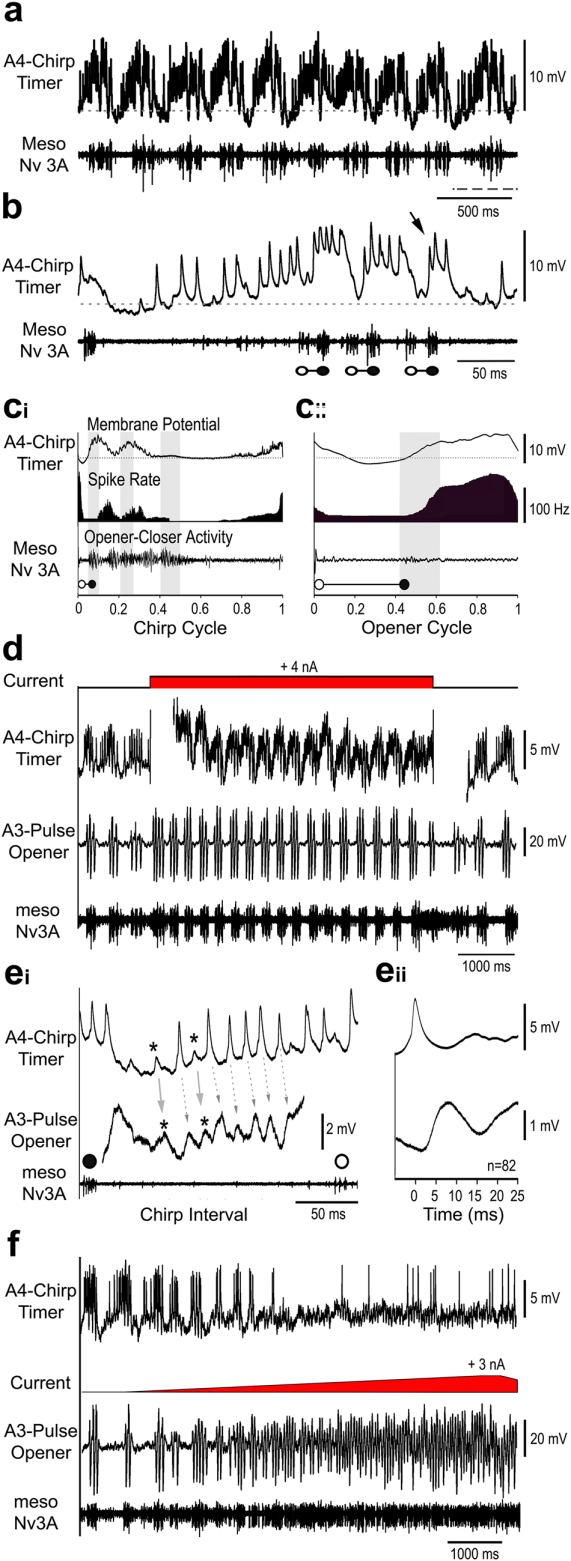
Activity of the *A4-Chirp-Timer*. **a** The interneuron spike activity is rhythmically coupled to the chirp pattern. **b** In the interchirp interval the neuron shows a gradual increase in its membrane potential and starts spiking well before the start of a chirp. It is depolarised throughout the chirp, spikes occur before the opener MN burst (open circles), and it is hyperpolarised before the closer activity (close circles). Grey dashed line represents the resting membrane potential. **c**_**i**_, **c**_**ii**_ Phase diagrams with the average membrane potential of the *A4-Chirp-Timer* (top), its average spike rate (middle) and the averaged meso-Nv3A activity (bottom) for the chirp cycle (**c**_**i**_) and opener cycle (**c**_**ii**_). Vertical grey bars indicate closer MN activity, and grey dashed line the resting membrane potential. *N * = 6 crickets, *n * = 60 chirps/240 pulses, AP = 970. **d** Paired recordings of the *A4-Chirp-Timer* and the *A3-Pulse-Opene*r. Depolarising current injection in the *A4-Chirp-Timer* with 4 nA for 5 s shortens the chirp period of fictive singing and increases the *A3-Pulse-Opener* membrane potential oscillations. **e**_**i,ii**_ Synaptic coupling between the *A4-Chirp-Timer* and the *A3-Pulse-Opener*, demonstrated during low spike activity by averaging the *A3-Pulse-Opener* membrane potential triggered by spikes of the *A4-Chirp-Timer* (*N * = 2, *n *  = 82). **f** A ramp of depolarising current with 3 nA injected in the *A3-Pulse-Opener* terminates the chirp pattern; it elicits rhythmic opener-closer cycles in the A3 neuron and a barrage of EPSPs in the *A4-Chirp-Timer*

The *A4-Chirp-Timer* showed very characteristic membrane potential changes during singing, with spike activity already occurring in the chirp intervals, and the maximum depolarisation and spike activity coupled to the chirps. The neuron was inhibited by 1–3 mV at the beginning of the chirp interval and did not spike for 52 ± 7.2 ms (*N* = 6; *n* = 60). Then a ramp depolarisation started about 77 ± 4.7 ms (*N* = 6; *n* = 60) before a chirp leading to an increasing spike activity (Fig. 6b), which at the beginning of the ramp was 53 ± 10.9 Hz, and increased to 140 Hz right at the start of a chirp (*N* = 6; *n* = 60) (Fig. 6 c_i_).

During a chirp, the activity of the *A4-Chirp-Timer* showed a maintained depolarisation modulated by rhythmic activity in phase with the pulse pattern. Rather broad spike bursts with 4–8 spikes preceded the opener activity by 26 ± 8 ms (*N * = 6; *n * = 60). The bursts were followed by a short inhibition, preceding the closer activity. Over a chirp, the depolarisation and also the spike activity gradually decreased (Fig. 6b,c_i_), the last depolarisation occurred with 1–3 spikes (Fig. 6b, arrow), thereafter the membrane potential dropped below the resting level.

The phase diagram over the chirp cycle (Fig. 6c_i_) shows the decrease in rhythmic spike activity over a chirp (phase 0.0 to 0.4) and also the build-up during the ramp depolarisation from phase 0.6 to 1.0. In the opener cycle (Fig. 6c_ii_) the *A4-Chirp-Timer* membrane depolarisation and spike activity were lowest at phase 0.1–0.4, before the start of the closer activity. The membrane depolarisation and spike activity then increased reaching a broad maximum at phase 0.6–0.9 with a mean spike rate of 125 Hz before the start of the opener burst. (Fig. 6c_ii_).

At times the fictive singing motor pattern was less regular, with low motoneuron activity and considerably extended chirp periods. Spike activity of the *A4-Chirp-Timer* still occurred in the long chirp intervals, but it was not modulated in a pulse pattern and was not coupled to singing motor activity. The spike rate of the bursts during low fictive singing was 62 ± 9 Hz (*N * = 3; *n * = 45), and significantly lower than during proper fictive singing (One-way ANOVA (*F*[2,6] = 7.57, *p * = 0.02).

### Paired intracellular recordings of the A4-Chirp-Timer and the A3-Pulse-Opener

Paired intracellular recordings of the *A4-Chirp-Timer* and the *A3-Pulse-Opener* (*N* = 5) allowed to check their functional connectivity (Fig. 6d–f). Injecting depolarising current (5 nA, 5 s) in the *A4-Chirp-Timer* dendrite during a sequence of weak and irregular fictive singing with a chirp period of 426 ± 92 ms induced a stable chirp pattern with 3–4 pulses/chirp, and decreased the chirp period to 362 ± 54 ms. During current injection also the rhythmic membrane potential oscillations of *A3-Pulse-Opener* became more prominent and the amplitude of its maximum depolarisation and inhibition in phase with the pulse pattern increased (Fig. 6d). The paired recordings revealed the synaptic coupling between the two interneurons (Fig. 6e_i_, e_ii_). In the chirp intervals spikes of the *A4-Chirp-Timer* preceded EPSPs in the *A3-Pulse-Opener* in a one-to-one manner. Averaging of the *A3-Pulse-Opener* membrane potential triggered by the spikes of the *A4-Chirp-Timer* (*N* = 2, *n* = 82) demonstrated 1.5 mV EPSPs starting after a latency of 1.5 to 2 ms and reaching a maximum amplitude after 7 ms (Fig. 6e_ii_) and indicate a direct synaptic connection between the *A4-Chirp-Timer* and the *A3-Pulse-Opener*. However, also subthreshold EPSPs with a similar delay occurred in both neurons (Fig. 6e, asterisks). The temporal coupling of spikes in the *A4-Chirp-Timer* and EPSPs in the *A3-Pulse-Opener,* therefore, could also have been caused by a common synaptic input, which first affected the neuron in A4 and then the *A3-Pulse Opener*.

When a current ramp rising to + 3 nA was injected in the *A3-Pulse-Opener* during fictive singing, the rhythmic chirp pattern in both neurons stopped (Fig. 6f). The membrane potential of the *A3-Pulse-Opener* rather started to oscillate in the pulse pattern and to drive opener-closer cycles. At the same time, the *A4-Chirp-Timer* became depolarised, it received a barrage of EPSPs, spiking only occasionally (Fig. 6f). Checking the membrane potential oscillations and spike activity of both neurons, we did not find evidence for a direct synaptic connection from the *A3-Pulse-Opener* to the *A4-Chirp-Timer.* However, as the *A4-Chirp-Timer* activity became coupled to the activity pattern of the *A3-Pulse-Opener* a polysynaptic feedback is indicated between the pulse pattern generating part of the singing-CPG and the chirp-timer network, at least the *A4-Chirp-Timer* neuron.

### Properties of the A5-Chirp-Timer neuron

**Fig. 7:**
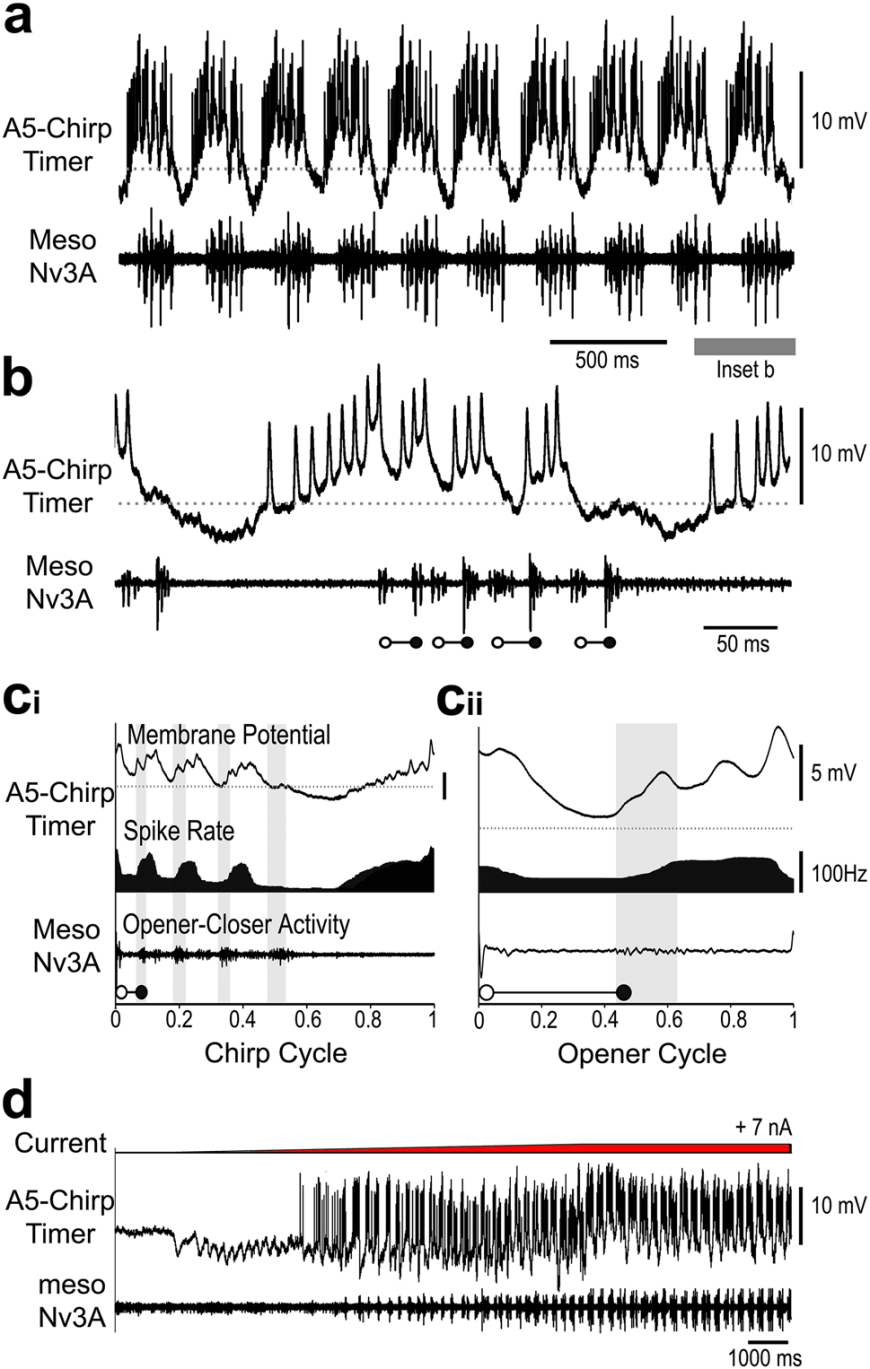
Activity of the *A5-Chirp-Timer*. **a** Spike activity is coupled to the generation of chirps. **b** The neuron is depolarised and starts spiking before the start of a chirp, a burst of spikes occurs before the opener activity (open circles), and the neuron repolarises before the closer activity (close circles). Grey dashed line indicates the resting membrane potential. **c**_**i**_, **c**_**ii**_ Phase diagrams with the average membrane potential (top), the average spike rate (middle) and the average meso-Nv3A activity (bottom) for the chirp cycle (**c**_**i**_) and opener cycle (**c**_**ii**_). Grey shaded bars indicate closer MN activity, grey dashed line represents the resting membrane potential. *N* = 2 crickets, *n* = 35 chirps/140 pulses, AP = 455. **d** Increasing current injection in the *A5-Chirp-Timer* with a ramp of 7 nA induced singing activity, with a transition between subthreshold membrane potential oscillations (left) to the production of chirps (right)

The *A5-Chirp-Timer* was recorded in two animals, it was not stained. Its activity pattern was very similar to the *A4-Chirp-Timer*, with an inhibition of 1–2 mV at the start of the chirp interval and a period without spikes for 66 ± 8.6 ms (*N* = 2, *n* = 22), followed a ramp in the build up to a chirp. Spike activity started at 81 ± 8.1 ms (*N* = 2; *n* = 22) before a chirp corresponding to phase 0.7 in the chirp cycle (Fig. 7c_i_), the discharge rate increased to 80–100 Hz at the beginning of the chirp.

During a chirp, the activity of the *A5-Chirp-Timer* showed a maintained depolarisation modulated by rhythmic activity in phase with the pulse pattern. Spike bursts with 3–4 spikes preceded the opener activity by 28 ± 3.1 ms (*N* = 2, *n* = 22), bursts were followed by an inhibition preceding the closer activity. As the overall depolarisation over a chirp gradually decreased (Fig. 7b), so did its spike activity preceding the opener MN activity (Fig. 7c_i_). The inhibition coupled to the pulses increased, as measured from the base of the last spike; to the first pulse it was 5.6 ± 0.4 mV, and to the last pulse it could go below the resting potential and was 6.4 ± 0.7 mV (*N* = 2, *n* = 22). After a chirp, the *A5-Chirp-Timer* could show either a weak depolarisation (Fig. 7b) or a spike burst, following the final closer activity.

The phase diagram for the chirp cycle (Fig. 7c_i_) shows a gradual depolarisation of the membrane and an increase in spiking at phase 0.7 within the interchirp interval, and a maximum discharge rate of about 100 Hz at phase 1/0 i.e. at the end/beginning of a chirp. The membrane potential oscillations and spike activity preceded the opener activity. Over the opener cycle (Fig. 7c_ii_) the membrane depolarisation and spike activity were lowest at phase 0.2–0.4 before the start of the closer activity. Activity then gradually increased reaching a broad maximum of spiking activity of about 100 Hz from phase 0.6–0.9 before the opener activity (Fig. 7c_ii_).

Injecting a depolarising current ramp rising to 7 nA in a resting cricket initiated singing (Fig. 7d). During the initial phase of current injection subthreshold rhythmic membrane potential oscillations occurred. When the current increased from 2.5 to 3.2 nA bursts of interneuron spikes preceded single opener-closer events. Further increase of the current to + 7 nA elicited singing motor activity, organised in chirps with 3–4 pulses.

### Activity and impact of the A5-Chirp-Start neuron

The *A5-Chirp-Start* neuron was recorded 8 times and stained 5 times with neurobiotin and 3 times with Alexa 568, no dye coupling was observed. Its dendritic arborisation in A5 looked very similar to the structure of the *A4-Chirp-Timer* and the *A3-Pulse-Opener* neurons, with symmetrically arranged dendrites along the midline of the ganglion. The anterior dendritic arborisation contralateral to the cell body was more elaborate. The axon was positioned very laterally in the contralateral connective; in ganglion A4 two collaterals project median and dorsal, forming arborisations on both sides along the midline (Fig. 1e). In two cases the axon could be traced towards T2, however further collaterals could not be revealed.

**Fig. 8:**
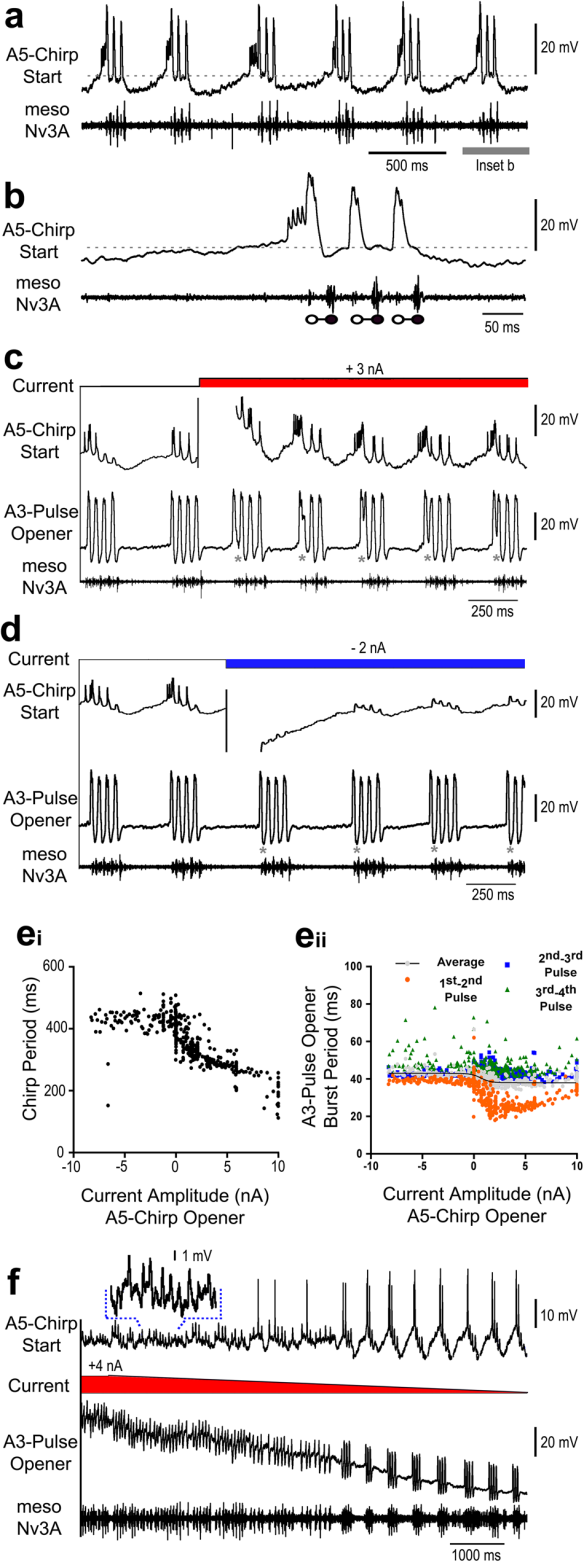
Activity of *A5-Chirp-Start* and impact on *A3-Pulse-Opener* activity. **a,b** During fictive singing the *A5-Chirp-Start* neuron shows a high frequency burst of spikes right before a chirp. **c,d** Double intracellular recordings of the *A5-Chirp-Start* and the *A3-Pulse-Opener*, depolarisation of the *A5-Chirp-Start neuron* increases the chirp period and alters the activity pattern of the *A3-Pulse-Opener* neuron, which prematurely terminates the first depolarisation and starts the second one. Asterisks indicate change in time course (**c**) of the first depolarisation and the amplitude of the first hyperpolarisation (**d**). **e**_**i**_ Effect of depolarising and hyperpolarising the *A5-Chirp-Start* neuron on the chirp period. **e**_**ii**_ Effect of depolarising the *A5-Chirp-Start* neuron on the *A3-Pulse-Opener* burst period. **f** An *A3-Pulse-Opener* ramp depolarisation decreasing from 4 nA is accompanied by the opener-closer MN activity in the pulse pattern and a transition to the chirp pattern as the current decreases. Activity in the *A5-Chirp-Start* neuron is coupled to the changing motor pattern. Inset shows section of the recording with higher resolution

The *A5-Chirp-Start* neuron showed a characteristic high-frequency spike burst at the beginning of a chirp, due to the timing of this activity we labelled it “chirp-start” interneuron (Fig. 8a, b). At the beginning of the interchirp interval the neuron was hyperpolarised, it then generated a gradual ramp-like depolarisation and at 38 ± 10 ms (*N* = 8, *n* = 80) before the start of a chirp it characteristically generated a plateau potential of 8–12 mV and a burst of 4–6 spike at 150 Hz. This was followed by a higher depolarisation of 23–27 mV with a burst of 2–3 spikes at a discharge rate of about 250 Hz, occurring 2.5 ± 0.5 ms before the opener and 26 ± 1.3 ms before the closer activity (*N* = 8; *n* = 80), this depolarisation was followed by an inhibition of 2–3 mV. The second depolarisation generated a discharge rate of about 200 Hz and repolarised towards the resting membrane potential, the third depolarisation came with a mean spike rate of 75 Hz, and after the chirp the membrane potential gradually dropped below the resting level (Fig. 8b).

During fictive singing, we simultaneously recorded the *A5-Chirp-Start* neuron together with the *A3-Pulse-Opener* (*N* = 3). Modulating the *A5-Chirp-Start* neuron with systematic de- and hyperpolarising current injections modulated the chirp pattern significantly (Fig. 8c,d), hyperpolarisation increased the chirp period from 381 ± 27 ms to 428 ± 41 ms, while depolarisation by 3 nA shortened it to 312 ± 58 ms (One-way ANOVA, *N* = 2, *n* = 483; *F*[2,480] = 251, *p* < .0001), and even 230 ms at higher depolarisation currents (Fig. 8e_i_).

Depolarising current injection in the *A5-Chirp-Start* neuron also had a significant impact on the activity of the *A3-Pulse-Opener* activity and altered the pattern of its first depolarisation at the start of a chirp (Fig. 8c, e_ii_). The first depolarisation of the *A3-Pulse-Opener* was terminated prematurely, the membrane potential dropped, but it did not reach the normal level of inhibition occurring between the bursts, and rather a second depolarisation was initiated. The amplitude of the first hyperpolarisation of the *A3-Pulse-Opener* - measured as the difference between the average resting membrane potential 80 ms before the start of a chirp and the peak of the inhibition, indicated by asterisks in Fig. 8c - significantly decreased (*F*[2,481] = 368, *p* < .0001). A post-hoc multi comparison revealed that during current injection the *A3-Pulse-Opener* membrane potential remained by 7.4 ± 9.3 mV above the resting potential (*N* = 2; *n* = 273, *p* < .0001), while it normally was hyperpolarised by -7.4 ± 2.8 mV below the resting potential (*N* = 2, *n* = 82). When hyperpolarising current was injected in the *A5-Chirp-Start* neuron the first hyperpolarisation of the *A3-Pulse-Opene*r at the beginning of a chirp increased significantly by 1–2 mV to -11.7 ± 1.1 mV (*N* = 2, *n* = 129; *p* < .0001), (Fig. 8d, asteriks).

Current injection in the *A5-Chirp-Start* also modulated the burst period between the first and the second *A3-Pulse-Opener* depolarisation. A post-hoc multiple comparison analysis revealed that depolarising current significantly decreased the first burst period from 38 ± 4 ms (*N* = 2, *n* = 82) to 28 ± 5 ms (*N* = 2, *n* = 273) (Fig. 8e_ii_, orange dots; *p* < .0001; pooled data for all positive current amplitudes), while hyperpolarising current did not change it (Fig. 8e_ii_, *p =* .55). Current injection had no effect on the period of the second and third *A3-Pulse-Opener* depolarisation (Fig. 8e_ii_, blue squares one-way ANOVA; *F*[2,460] = 5.1, *p =* .007). A significant effect of the current injection on the period between the third and the fourth depolarisation was revealed with a one-way ANOVA (Fig. 8e_ii_, green triangles; *F*[2,420] = 19.8, *p* < .0001), during hyperpolarisation of the *A5-Chirp-Start* neuron the period was slightly extended from 44 ± 2.4 ms to 48 ± 6.2 (pooled data; *p* < .0001). In addition, hyperpolarizing current increased the variability of period between third and fourth depolarisation, no effect occurred during depolarising current injection (*p =* .61).

Depolarising current injection into the *A3-Pulse-Opener* with a decreasing ramp initially elicited the typical membrane potential oscillations coupled to the pulse pattern (Fig. 8f), which was reflected in the *A5-Chirp-Start* activity pattern. When the injected current decreased, in both neurons the chirp pattern became established, indicating a transition between an activity pattern dominated by the *A3-Pulse-Opener* neuron to a pattern determined by the singing-CPG network. Due to the structure of the neurons, the *A5-Chirp-Start* neuron cannot directly couple to the pulse pattern imposed by the *A3-Pulse-Opener*, therefore, a feedback loop seems to alter the activity of the chirp timer network.

Paired recordings (*N* = 2) were also obtained between the *A5-Chirp-Start* neuron and the *A4-Pulse-Opener*, current injection into the *A5-Chirp-Start* neuron did not reveal any changes in the *A4-Pulse-Opener* activity.

### Interneurons with spike activity in the interchirp interval, and rhythmic inhibition during the chirps

In each of the abdominal ganglia A5 and A6 we recorded and identified an interneuron which spiked during the interchirp interval and received rhythmic inhibition during the chirps. The structure of the neuron in A6 was revealed twice with neurobiotin, each staining labelled one cell body and extensive dendrites in A6, similar to the symmetrical arborisation patterns reported before. On each side of the midline, the primary neurite gave off two extensive anterior projecting dendrites, the one on the ipsilateral side being more elaborate (Fig. 1f), and smaller dendrites projecting posteriorly. The ascending axon ran laterally in the connective, its anterior projections were not resolved.

**Fig. 9:**
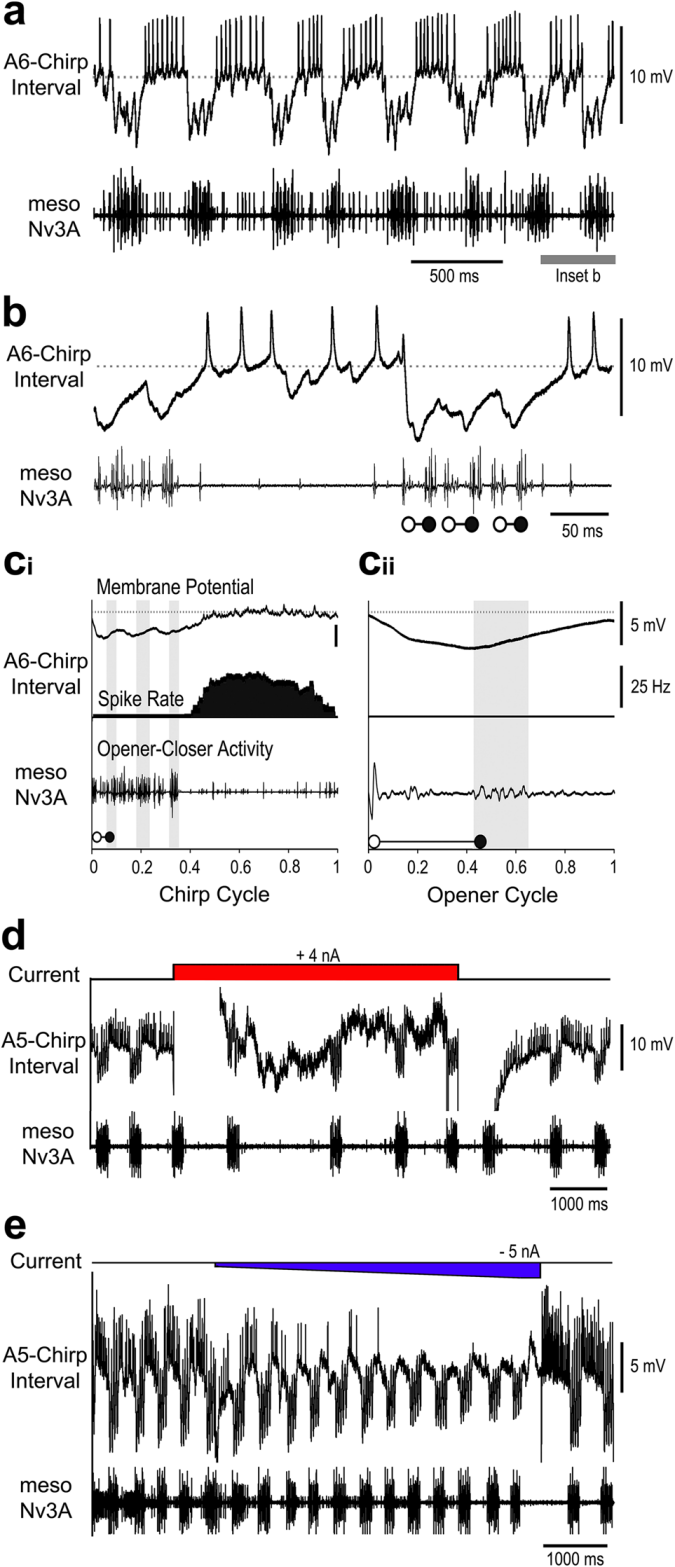
Activity of *A5-Chirp-Interval* and *A6-Chirp-Interval* neurons. **a,b** During fictive singing the neurons spike in the interchirp intervals and are rhythmically inhibited during the chirps. **c**_**i**_, **c**_**ii**_ Phase diagrams with the averaged membrane potential (top), the average spike rate (middle) and the average meso-Nv3A activity (bottom) for the chirp cycle (**c**_**i**_) and opener cycle (**c**_**ii**_). *N* = 2 crickets, *n* = 35 chirps/105 pulses, AP = 235. Grey shaded sections indicate closer MN activity, grey dashed line indicates the resting membrane potential. **d** Depolarisation of the *A5-Chirp-Interval* neuron increases the chirp period. **e** Hyperpolarisation of the *A5-Chirp-Interval* neuron reduces background activity in the meso-Nv3A recording and decreases the chirp period

The A5 and A6 interneurons showed very similar activity patterns, and were labelled *A5-* and *A6-Chirp-Interval* neurons. The neurons were depolarised during the interchirp interval and spiked with a rate of 25–50 Hz (Fig. 9a,b). In phase with the fictive chirps, the activity of both neurons was dominated by inhibitory postsynaptic potentials (IPSPs) coupled to the pulse pattern (Fig. 9b). The inhibition started about 2 ms before the opener activity, it reached its peak of 5–8 mV after the opener burst, and then declined during the closer activity before the next inhibition started.

The phase diagrams for the chirp-cycle revealed the broad coupling of the depolarisation and a broad maximum of 30 Hz mean spike activity in the chirp interval, whereas the inhibition occurred in phase with the opener-closer activity (Fig. 9c_i_). The phase diagram over the opener cycle revealed the inhibition occurring after the opener burst, in the interval between the opener and closer activity. (Fig. 9c_ii_).

Modulating the activity pattern of the *A5-Chirp-Interval* neuron by intracellular current injection altered the singing pattern (Fig. 9d). Depolarising current pulses (4 nA, 5 s) increased spiking activity and shifted the median chirp duration from 160 ms (156 ms/171 ms) to 166 ms (161 ms/199 ms), (*N* = 2, *n* = 36). The effect on the chirp period was more pronounced, it increased from 475 ms (416 ms/551 ms), (*N* = 2, *n* = 104) to 526 ms (457 ms/661 ms), (*N* = 2, *n* = 36). A hyperpolarising current ramp (from 0 to -5 nA, 5 s) was applied during instable fictive singing (Fig. 9e). It gradually suppressed spike activity, and at the same time the singing motor pattern became more clearly expressed as background activity decreased. Upon the release from hyperpolarisation the interneuron’s discharge rate transiently increased to 110 Hz, and at the same time the interchirp interval became extended to 880 ms (Fig. 9e). The effect of these experiments indicates that increased spiking activity in the *A5-Chirp-Interval* interneurons will have an inhibitory effect on singing. Current injections in the *A6-Chirp-Interval* neuron were not tested. A further interneuron was recorded twice in the A6 ganglion with spike activity during the chirp intervals and rhythmic depolarisations coupled to the pulses, it was not stained or tested for any impact on singing activity.

## Discussion

Chirping crickets such as *G. bimaculatus* combine a 30 Hz pulse pattern with a 3 Hz chirp pattern during calls. We analysed the underlying network organisation by recording abdominal singing-interneurons, which control the timing and coordination of the two motor patterns.

### Structure and organisation of singing-interneurons

This was the first study of the singing network along the abdominal ganglion chain, and although the description of interneurons will not be complete, key elements of the system have been revealed. The identified singing-interneurons share some general morphological features such as the position of the cell body, the projection of the main neurite, the symmetrical pattern of the dorsally located dendrites and a contralateral ascending axon with collaterals in the thoracic ganglia (Fig. 1). For the *A3-Pulse-Opener* (Schöneich and Hedwig 2011, 2012) these characteristic features are conserved over cricket clades (Jacob and Hedwig 2019), here we reveal that they are also characteristic for the singing-interneurons along the abdominal ganglion chain.

Dye coupling, indicating gap-junctions between neurons, occurs between the left and right *A3-Pulse-Opener* (Schöneich and Hedwig 2012), and the *A4-Chirp-Timer*, and will contribute to synchronise of the activity of bilateral sibling neurons. As the cricket singing motor pattern is based on strictly coupled and coordinated movements of both front wings, a laterally specific processing of the motor signals may not be required. Also the bilateral dendritic arborisations of the singing interneurons will integrate input from both sides of the CNS, and thoracic axon collaterals of chirp-timer neurons project across the midline, forwarding activity to both sides of the network.

The singing-neurons along the abdominal ganglion chain demonstrate a distributed network, in line with the conclusions drawn from lesioning the abdominal CNS (Schöneich and Hedwig 2011; Jacob and Hedwig 2016). These experiments point towards a modular organisation, with the posterior ganglia A4–A6 housing a chirp-timer and the anterior ones A3 and A4 housing a pulse-timer network. This modular dissociation of a chirp-timer network and a pulse-timer network shares similarities with networks in other acoustic communicating species. In anurans, the chirp-timer interneurons code the two temporal parameters (pulses and chirps), they however only modulate the chirp pattern (Zornik and Yamaguchi 2012). A similar dissociation of functions is observed in toadfish, where separate hindbrain nuclei control either the song duration or the pulse frequency (Chagnaud et al. 2011). A modular organisation of motor networks is also proposed for the crayfish swimmeret system (Smarandache et al. 2009; Mulloney and Smarandache-Wellmann 2012) with independent swimmeret-CPGs in the abdominal ganglia, and for the control of leg movements in insects based on the cooperation of modular CPGs (Bidaye et al. 2018).

### Pulse-timer network

Neurons generating the pulse pattern showed a strict temporal coupling to the MN bursts, they did not spike during the interchirp interval while their membrane potential showed a subthreshold ramp depolarisation before a chirp. Crucially, depolarisation of either the *A3-* or *A4-Pulse-Opener* (Figs. 2 and 4) reliably elicited an ongoing sequence of opener-closer activity, which was not structured in the chirp pattern. Simultaneous intracellular recordings of the *A3-* and *A4-Pulse-Opener* showed similar membrane potential oscillations but did not reveal an excitatory synaptic coupling between both neurons (Fig. 5). The presence of the *A4-Pulse-Opener* may indicate a redundant organisation of the network, this may contribute to stabilise the motor output, and explain why crickets still can generate the pulse pattern, after hemisection of the A3 ganglion (Jacob and Hedwig 2016).

The pulse-closer neurons showed a characteristic rhythmic inhibition preceding the opener burst, while their depolarisation and spike activity was coupled to the closer MN activity. Releasing the pulse-closer neurons from inhibition triggered a post-inhibitory rebound (PIR) and spike activity, while depolarisation did not elicit singing activity. These properties correspond to the closer interneurons in the A1 and A2 neuromeres (Schöneich and Hedwig 2012). Our data support the suggestion that reciprocal inhibitory coupling of “half-centre oscillators”, i.e. the pulse-opener and pulse-closer interneurons, generates the rhythmic, alternating opener-closer activity underlying the pulse pattern (Bentley 1969; Schöneich and Hedwig 2012). In such a network a neuron escapes from inhibition due to its intrinsic membrane properties generating a post-inhibitory rebound and spike activity. In turn it will inhibit the other half-centre and together they generate a rhythmic activity pattern as long as the system is driven by a tonic input (Perkel and Mulloney 1974; Satterlie 1985a; Friesen 1994, Marder and Bucher 2001).

The existence of both pulse-opener and pulse-closer interneurons in A3 and A4 is consistent with a modular pulse-timer network in these ganglia. It demonstrates, that activity driving the complete opener-closer cycle is assembled in the anterior abdominal ganglia and forwarded towards the mesothoracic motoneurons. A pattern generating singing-network in the mesothoracic ganglion is not required; and it may not be present as hemisection of the ganglion does not impede singing motor activity (Hennig and Otto 1996). Earlier suggestions on the minimal CNS structures controlling singing in crickets (Huber 1962), will need to be revised. The rhythmic activity of the pulse-timer network depends on an excitatory synaptic input from the chirp-timer network. It will stop, once this excitatory drive ceases, for example, if cercal wind stimuli cause a transient inhibition (Jacob and Hedwig 2015).

### Generation of the chirp pattern

Neurons of the chirp-timer network originated in ganglia A4 and A5. They showed a suprathreshold ramp-depolarisation in the interchirp interval and a built up of spike activity tens of milliseconds before the start of a chirp. Importantly, upon depolarising current injection the *A4-* and the *A5-Chirp-Timer* neurons induced the complete chirp pattern. The properties of the chirp-timer interneurons are congruent with data from lesioning experiments (Jacob and Hedwig 2016). Cutting the connectives either between A4 and A5 or A5 and A6, and splits of the A5 ganglion, which completely or partially removed chirp-timer neurons from the network, all had an impact on the chirp pattern and increased the chirp duration and period. The chirp-timer interneurons seem to deliver an excitatory drive to the pulse-timer network. Simultaneous recordings between the *A4-Chirp-Timer* and the *A3-Pulse-Opener* reveal a synaptic connection and flow of excitation from the chirp-timer network to the pulse-timer network (Fig. 6). A posterior to anterior flow of activity is also given by the effect of depolarising current injection in the *A5-Chirp-Start* neuron, which substantially altered the activity of the *A3-Pulse-Opener* neuron (Fig. 8).

The ramp-depolarisation of the chirp-timer interneurons, indicates these neurons are driven by a gradual synaptic input (Long et al. 2010) and/or that intrinsic changes in membrane conductance lead to a gradual depolarisation. Long-lasting ramp depolarisation is fundamental for the initiation and maintenance of the swimming behaviour in Tritonia (Lennard et al. 1980; Getting and Dekin 1985; Frost et al. 2001) and the initiation of swimming in leeches (Brodfuehrer and Friesen 1986). In crickets such as *G. bimaculatus* the main candidates to drive the singing-network are the bilateral calling song command neurons, which descend from the brain. They elicit singing upon intracellular stimulation and increase the chirp rate with increasing spike activity (Hedwig 2000). Additionally, the ventilation-CPG and the singing-CPG are coupled by feedforward excitation, and ventilation can provide an excitatory input to the singing-network timed to the chirp rhythm (Schöneich and Hedwig 2019).

Hints on possible intrinsic properties of the chirp-timer neurons supporting the generation of the chirp pattern may come from lesion experiments (Kutsch and Otto 1972). When the cervical connectives in *G. campestris* males were cut, after several days they spontaneously generated short bouts of calling song activity. In this situation the abdominal singing-network is sufficient to generate the motor pattern without commands from the brain, however, a synaptic reorganisation of the system may have to be considered. If the ramp-depolarisations are supported by intrinsic properties, inward currents with slow kinetics matching the chirp period of 300–500 ms will be of special importance (Harris-Warrick 2010). In brainstem neurons of the dorsal and medullary raphe, currents that modulate the ramp-depolarisation phase of these neurons include the transient-A-type K^+^ (I_A_) current (Aghajanian 1985; Segal 1985), the Ca^2+−^activated K^+^ current (IKCa), and the depolarisation-activated cation current (Penington and Kelly 1993). Information on conductances in the cricket singing-interneurons are not yet available. In preliminary tests exposing the abdominal ganglia to ZD-7288, a blocker of HCN channels, we could not reveal an effect on the singing motor pattern.

The *A5-* and *A6-Chirp-Interval* neurons received a rhythmic inhibition coupled to the pulse pattern, while spike activity occurred in the interchirp interval. Increased spike activity of the neurons had an inhibitory effect and increased the chirp period. This is similar to the impact of ventilation neurons descending from the suboesophageal ganglion (Otto and Janiszewski 1989; Otto and Hennig 1993). During singing, these interneurons receive IPSPs coupled to the pulse pattern, and depolarising current injection increases the chirp period.

### Feedback loops

All chirp-timer neurons were rhythmically modulated in the pulse pattern, however, all interganglionic singing-neurons have an ascending axon and, therefore, cannot directly provide feedback to posterior ganglia. Initiating the pulse pattern by depolarising the *A3-Pulse-Timer*, however, coupled the posterior chirp-timer neurons to the pulse pattern. Feedback to the chirp-timer neurons may be provided by anterior descending pathways, like the descending opener interneuron in T3, with its dendrites in the metathoracic ganglion, an axon projecting through all abdominal ganglia, and spikes coupled to the pulse pattern. Upon depolarisation, the metathoracic descending opener interneuron elicits singing, and could be effective by activating the abdominal singing-neurons (Schöneich and Hedwig 2012).

Inhibitory feedback may be provided by other descending interneurons such as the suboesophageal interneurons, which increase the chirp period upon intracellular depolarisation (Otto and Janiszewski 1989; Otto and Hennig 1993). A likely candidate for inhibitory feedback is the corollary discharge interneuron, which inhibits the auditory pathway in phase with the closer activity (Poulet and Hedwig 2006). The neuron also projects along the whole abdominal ganglion chain and could inhibit the chirp-timer and the chirp-interval neurons in phase with the pulse pattern.

### Why this organisation of the singing network?

Our data suggest a flow of activity in the abdominal singing-network from posterior to anterior, and functionally the opposite would hardly make sense. The ramp-depolarisation and spike activity in the chirp-timer neurons starts tens of millisecond before a chirp, whereas the pulse-timer neurons show no spikes in the interchirp interval. Furthermore, when considering the timing between interneuron spikes and opener MN activity a decrease in latency from posterior to anterior is apparent; the *A5-Chirp-Timer* and the *A4-Chirp-Timer* precede the MN by 28 ± 3.1 ms and 26.8 ± 8 ms, respectively, while the *A4-Pulse-Opener* and the *A3-Pulse-Opener* activity occur with a latency of 17.9 ± 1.9 ms and 10.4 ± 1.2 ms, respectively. Also the effect of depolarising the *A5-Chirp-Start* neuron (Fig. 8), and the *A4-Chirp-Timer* neuron (Fig. 6) demonstrate a flow of activity from posterior to anterior. When passing from posterior to anterior along the chain of abdominal ganglia the neuronal activity underlying the singing motor pattern is progressively shaped and fine-tuned from a chirp-pattern to a pulse-pattern occurring in the chirp rhythm.

In adult insects abdominal segments bear no appendages, and not very much is known about the contribution of the unfused abdominal ganglia to rhythmic motor patterns, besides their involvement in generating the abdominal ventilatory pumping movements (Huber 1962; Miller 1966), being involved in crawling movements in fly larvae (Pulver et al. 2015), while in crustacean the abdominal ganglia generate the rhythmic swimmeret movements (Mulloney and Smarandache-Wellmann 2012). All these motor patterns have in common a wave of excitation flowing from the posterior abdominal ganglia towards the anterior. In the locust flight system serial homologous flight interneurons in anterior abdominal ganglia have been indicated to reflect an evolutionary earlier stage of the system (Robertson et al. 1982). In a similar way, the abdominal organisation of the cricket singing-network may indicate that an evolutionary old motor system has been adapted for song pattern generation. Its modular organisation along the abdominal ganglia may allow for combining neuronal components in different ways to generate the variety of song pattern encountered in extant cricket species (Alexander 1962; Jacob and Hedwig 2019).

## Abbreviations

CPG: Central Pattern Generator
PIR: Post-inhibitory rebound
T2,T3: Meso, metathoracic ganglion
A1-A6: Abdominal ganglia 1 to 6
Nv: Nerve
MN: Motoneuron
EPSP: Excitatory postsynaptic potential
